# Saturated fatty acid concentrations are predictive of insulin sensitivity and beta cell compensation in dogs

**DOI:** 10.1038/s41598-024-63373-5

**Published:** 2024-06-02

**Authors:** Matthew Peloquin, Ashley Tovar, Jessica L. Graves, Darko Stefanovski, Katya Tucker, Entonio Marietti, Karen Greenwood, Celine-Lea Halioua-Haubold, Dina Juarez-Salinas

**Affiliations:** 1 Cellular Longevity Inc., San Francisco, CA USA; 2 Department of Clinical Studies - NBC, University of Pennsylvania School of Veterinary Medicine, Kennett Square, PA USA

**Keywords:** Ageing, Metabolism, Animal physiology

## Abstract

Chronic feeding of a high fat diet (HFD) in preclinical species induces broad metabolic dysfunction characterized by body weight gain, hyperinsulinemia, dyslipidemia and impaired insulin sensitivity. The plasma lipidome is not well characterized in dogs with HFD-induced metabolic dysfunction. We therefore aimed to describe the alterations that occur in the plasma lipid composition of dogs that are fed a HFD and examine the association of these changes with the clinical signs of metabolic dysfunction. Dogs were fed a normal diet (ND) or HFD for 12 weeks. Insulin sensitivity (S_I_) and beta cell compensation (AIR_G_) were assessed through an intravenous glucose tolerance test (IVGTT) and serum biochemistry was analyzed before the introduction of HFD and again after 12 weeks of continued ND or HFD feeding. Plasma lipidomics were conducted prior to the introduction of HFD and again at week 8 in both ND and HFD-fed dogs. 12 weeks of HFD feeding resulted in impaired insulin sensitivity and increased beta cell compensation measured by S_I_ (ND mean: 11.5 [mU/l]^–1^ min^–1^, HFD mean: 4.7 [mU/l]^–1^ min^–1^) and AIR_G_ (ND mean: 167.0 [mU/l]min, HFD mean: 260.2 [mU/l]min), respectively, compared to dogs fed ND over the same duration. Chronic HFD feeding increased concentrations of plasma lipid species and deleterious fatty acids compared to dogs fed a ND. Saturated fatty acid (SFA) concentrations were significantly associated with fasting insulin (R^2^ = 0.29), S_I_ (R^2^ = 0.49) and AIR_G_ (R^2^ = 0.37) in all dogs after 12 weeks, irrespective of diet. Our results demonstrate that chronic HFD feeding leads to significant changes in plasma lipid composition and fatty acid concentrations associated with metabolic dysfunction. High SFA concentrations may be predictive of deteriorated insulin sensitivity in dogs.

## Introduction

Insulin sensitivity is the ability of multiple organ systems to respond to insulin and effectuate physiological processes efficiently, such as initiating glucose uptake into muscle or inhibiting lipolysis in adipose tissue. Failure to maintain adequate insulin sensitivity and action, commonly referred to as insulin resistance, is a hallmark of various metabolic diseases in humans, such as metabolic syndrome^1^ (MetS), type II diabetes^2^ (T2D) and non-alcoholic fatty liver disease^1^. Moreover, advancing chronological age also results in broad metabolic decline that is often associated with the onset of disease, marked by decreased insulin sensitivity and impaired insulin clearance in rodents^3,4^, dogs^5,6^ and humans^7,8^.

Preclinical rodent models that induce metabolic dysfunction, such as chronic high fat diet (HFD) feeding, are commonly used to investigate aspects of various metabolic diseases. In mice, HFD feeding reproducibly leads to body weight gain, adipose expansion, dyslipidemia, hyperglycemia and hyperinsulinemia^9^. Consequently, excess circulating insulin and fatty acids contribute to lipid deposition in the liver and muscle, systemic and local inflammation and impairments to insulin sensitivity in peripheral tissues^10,11^. The use of these rodent models has been integral to the mechanistic understanding and subsequent therapeutic development for treating metabolic disease.

Although large animal models of metabolic dysfunction are less commonly employed, dogs are an excellent model of human disease due to their complex physiology, exposure to the same environmental factors and similarities in their timeline of disease onset. Many outcomes from studies using dogs have yielded similar phenotypes to that seen in rodent studies and naturally occuring metabolic disease. Feeding dogs a HFD leads to body weight gain, primarily driven by the expansion of adipose tissue, with accompanying fasting hyperinsulinemia and impaired insulin sensitivity^12–14^. This adipose expansion in dogs, specifically the hypertrophy of the visceral adipocytes, has been shown to correlate with deteriorated insulin sensitivity—A phenotype previously described in human populations^12,15^. Moreover, HFD-fed dogs experience elevations in clinically measured lipids, such as total triglycerides and cholesterol, producing a biochemical profile indicative of broad metabolic dysfunction^16^.

However, much less is known about changes in plasma lipid composition in metabolically stressed dogs^17,18^. Investigations in young, healthy dogs have revealed a potentially causal role of certain fatty acids in deteriorating insulin sensitivity. While not all fatty acids and lipid species negatively impact insulin sensitivity, co-infusing a neutral lipid and fatty acid cocktail comprised of linoleic, oleic, palmitic, linolenic and stearic acid at various time points during a hyperinsulinemic euglycemic clamp resulted in blunted glucose uptake into the muscle, altered endogenous glucose production by the liver and impaired whole body insulin action^19^. Although infusing these fatty acids is sufficient to produce an insulin resistant phenotype, it is still unclear what effect HFD feeding has on the plasma lipidome and if these changes are related to insulin sensitivity and action in dogs.

In this investigation, we aimed to further characterize several aspects of the HFD dog model: First, we aimed to characterize any HFD-induced changes in plasma lipid composition, specifically focusing on fatty acid species. Second, we aimed to recapitulate previous studies which demonstrate deteriorated insulin sensitivity and hyperinsulinemic beta cell compensation resulting from HFD feeding. Lastly, we aimed to determine if any association exists between the changes in lipid composition and insulin sensitivity and beta cell compensation in dogs.

## Methods

### Dogs

Male mixed-breed dogs and beagles (7–8 mixed-breed/group + 3 beagles/group) aged 4–7 years old, between 13 and 26 kg in weight, a BCS score of 4–6, and of mixed castration status (4–5 castrated/per group) were supplied by ClinVet of ClinGlobal (South Africa). Descriptive statistics of the groups pre-study enrollment is reported in Supplemental Table 1. All animal studies were submitted to and approved by the ClinVet (South Africa) Institutional Animal Care and Use Committee. Certificates of approval were issued. An ethics approval certificate was issued under National Health Research Ethics Council Reg No. AREC-230817–020. The study plans were designed to allow the use of the study animals in compliance with the ClinVet policy on the ethical use of animals, using the most recent version South African National Standard SANS 10386: *The care and use of animals for scientific purposes*, as a reference^20^. All methods were performed in accordance with the relevant guidelines and regulations. Dogs enrolled in this study received regular blood draws and examinations. No other procedures were conducted during this study. All dogs recovered and were returned to the ClinVet colony upon study completion. This study is reported in accordance with ARRIVE guidelines.

### Study design

**Figure 1:**
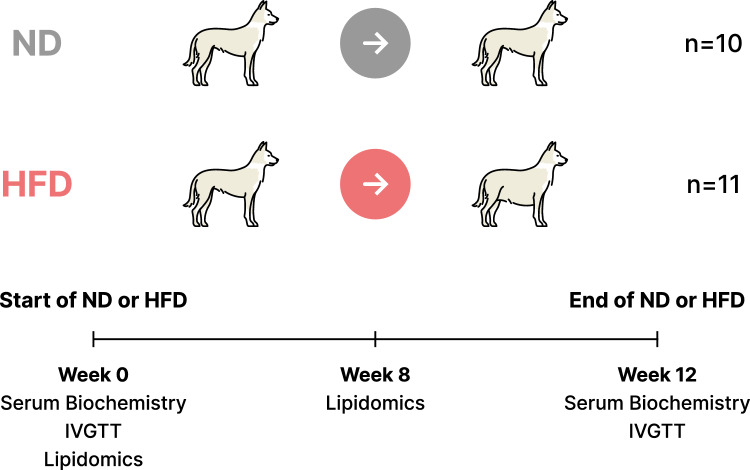
Experimental design. Serum biochemistry and IVGTT were assessed in dogs at week 0 (pre-HFD) and again after 12 weeks of the HFD or ND feeding (Week 12). Lipidomics were assessed at week 0 and after 8 weeks of the HFD or ND feeding (Week 8).

Prior to study start, all dogs were fed a dry normal diet (ND) which contained 55% of total calories derived from fat (VetsBrands Premium adult maintenance dog food, manufactured by VetsBrands; Reg No.: V 24369). The daily food ration for each dog was determined by body weight at study start and following the Vetbrands recommended guidelines for grams of food per kilogram of body weight. Dogs were randomized into ND or HFD groups balanced across age and body weight. On Day 0, dogs were randomized into the HFD group (n = 11) were transitioned from the aforementioned ND to a 66% (total calories) fat diet for 7 days and then given a 74% (total calories) fat diet (HFD) through the remainder of the study, for a total duration of 12 weeks. Increased dietary fat content was derived from the incorporation and thorough mixing of wet food (Husky Adult [Chunky], manufactured by Purina; Reg No.: V13339) and pork lard to the ND. Dogs in the ND group (n = 10) were continued on the dry ND through the conclusion of the study. Dogs assigned to a HFD were offered double the daily ration of food as the ND to maximize opportunity for consumption. Food intake was quantified by weighing the amount of diet presented subtracted from the amount of diet remaining, with lard pre-incorporated, if applicable. Body weights were assessed at study enrollment and again weekly over the 12-week duration. Study design details are described in Fig. 1. Macronutrient composition and dietary details for all diets used are reported in Supplement Tables 2, 3.

### IVGTT

Intravenous glucose tolerance tests (IVGTT) were performed at week 0 (prior to HFD initiation) and week 12, and were carried out as previously described^15^. Briefly, dogs were fasted overnight prior to conducting the procedure. Blood was sampled and serum and plasma separated at − 10 and − 1 min pre-glucose bolus to establish fasting glucose and insulin values. At 0 min (t = 0), a glucose bolus (0.3 g/kg, i.v.) was delivered. At t = 20 min a bolus of insulin (0.03 U/kg, i.v.) was delivered. Blood was collected at 2, 4, 6, 10, 14, 19, 22, 30, 40, 50, 60, 80, 100, 120, 150, and 180 min post-glucose bolus. Serum was then analyzed for glucose and plasma was analyzed for insulin levels at each time point. In a blinded fashion, glucose and insulin values were entered into the MINMOD Millenium 6.02 software to generate parameter estimates for insulin sensitivity (S_I,_), and glucose effectiveness (S_G_) for each animal. Acute insulin response to glucose (AIR_G_) was calculated from the AUC of insulin from t = 0 to t = 10 and the disposition index (DI) was calculated by the multiplication of AIR_G_ and S_I_.

A single dog in the ND group was removed as an outlier due to a supraphysiological S_I_ value (44.2 [mU/l]^–1^ min^–1^) at week 12.

### Serum biochemistry

Measures of leptin, non-esterified fatty acids (NEFA), triglycerides, cholesterol, aspartate aminotransferase (AST), alanine transaminase (ALT), alkaline phosphatase (ALP), creatine kinase (CK) were derived from fasting blood samples which were serum separated for analysis at week 0 (prior to diet initiation) and week 12. Serum canine leptin and adiponectin concentrations were measured by single-analyte enzyme-linked immunosorbent assay (ELISA) (Canine Leptin ELISA, Millipore EZCL-31 K and Adiponectin ELISA (MBL International CY-8052: CircuLex Dog, respectively). Serum NEFA concentrations were measured using the Cobas 6000 Chemistry Analyzer (Charles River Laboratories, Spencerville, Ohio). Serum clinical chemistry, fasting glucose, and fasting insulin quantification was performed and analyzed using the Abbott Alinity chemistry analyzer by PathCare (South Africa).

### Lipidomics

Targeted lipidomic profiling was conducted at Metabolon Inc (Complex Lipids Targeted Panel; Morrisville, NC) on canine plasma prior to study start (week 0) and again at 8 weeks post diet intervention (week 8). Lipids were extracted from plasma in the presence of deuterated internal standards using an automated BUME extraction according to the method of Löfgren et al.^21^. The extracts were dried under nitrogen and reconstituted in ammonium acetate dichloromethane:methanol. The extracts were transferred to vials for infusion-MS analysis, performed on a Shimadzu (Pleasanton, CA) LC with nano PEEK tubing and the Sciex (Toronto, Canada) SelexIon-5500 QTRAP. The samples were analyzed via both positive and negative mode electrospray. The 5500 QTRAP was operated in MRM mode with a total of more than 1100 MRMs. Individual lipid species were quantified by taking the ratio of the signal intensity of each target compound to that of its assigned internal standard, then multiplying by the concentration of internal standard added to the sample.

Lipid class concentrations were calculated from the sum of all molecular species within a class, and fatty acid compositions were determined by calculating the proportion of each class comprised by individual fatty acids. Lipid classes quantified are: Ceramides (CER), cholesteryl esters (CE), diacylglycerol (DAG), dihydroceramide (DCER), hexosylceramide (HCER), lactosylceramide (LCER), lysophosphatidylcholine (LPC), lysophosphatidylethanolamine (LPE), monoacylglycerol (MAG), phosphatidylcholine (PC), phosphatidylethanolamine (PE), phosphatidylinositol (PI), sphingomyelin (SM), triacylglycerol (TAG). All lipid classes and species measured by the Complex Lipids Targeted Panel from Metabolon are reported in Supplemental Table 4.

### Statistical analysis

Descriptive analyses included computation of means, standard deviations, medians, interquartile ranges (IQR) of continuous variables and tabulation of categorical variables. Tests of normal distribution (Shapiro–Wilk) were performed to determine the extent of skewness of the data. Frequency counts and percentages were used for summarizing categorical data.

Differences in body weight and food consumption were analyzed using mixed effects models using diet and week as main and interaction effects. Post-hoc group comparisons were performed to estimate group differences at each timepoint. Group differences in percent change in body weight and food consumption were analyzed using Two-sample t-tests at each timepoint. IVGTT parameters were estimated using statistical package STATA 17MP (StataCorp; College Station,TX) as previously described^18^. Two-sample *t*-tests were used to test group differences in lipidomics, serum biochemistry, and IVGTT parameters. Data are expressed as mean ± standard deviation. Pearson’s correlations were used to explore simple correlations between fatty acid species, fasting insulin, S_I_ and AIR_G_ at the conclusion of the study across both diet types. All statistical tests were two-sided with an alpha-level of 0.05 and were performed in R version 4.1.2. All subjects were included in analyses (ND = 10, HFD = 11) with the exception of data pertaining to the IVGTT (N = 9, HFD = 11) as described in the IVGTT methods section.

## Results

### Body weight and food intake after 12 weeks of ND or HFD

**Figure 2:**
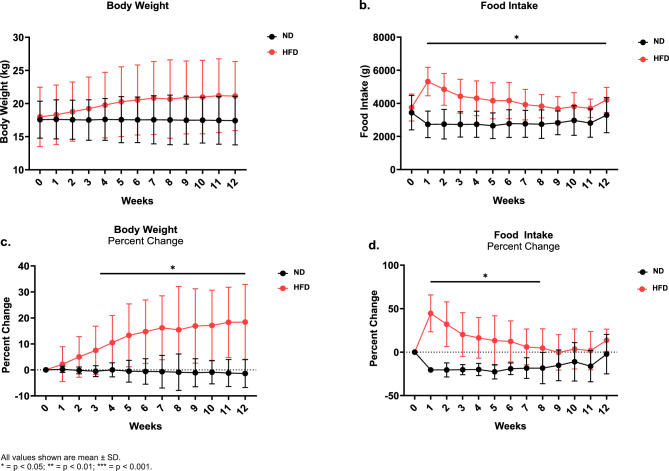
(**a**) Body weight and (**b**) food intake in dogs fed a ND (n = 10) or HFD (n = 11) for 12 weeks. Percent change from week 0 in (**c**) body weight and (**d**) food intake, in dogs fed a ND (n = 10) or HFD (n = 11) for 12 weeks. All values shown are mean ± SD. *p < 0.05; **p < 0.01; ***p < 0.001.

At the start of the study (week 0), dogs in both ND and HFD groups were similar in body weight and food intake (Fig. 2a,b). After the 1st week, dogs in the HFD group consumed significantly more food per week on average than dogs fed ND (5315.9 ± 859.5 g, 2729.6 ± 801.8 g, p < 0.001), which continued through all 12 weeks of the study (Fig. 2b). The average body weights of ND-fed dogs remained stable throughout the duration of the study.

Similarly, by week 1, HFD-fed dogs had a significantly greater percent change in food intake compared to dogs fed ND (HFD: 44.6 ± 21.2%, ND: − 20.5 ± 3.0%, p < 0.001), which continued to week 8 (Fig. 2d). By week 9, the body weight of dogs fed HFD tended to increase as compared to ND-fed dogs, though this change was not significant (HFD: 20.9 ± 5.5 kg, ND: 17.5 ± 3.6 kg, p = 0.09) (Fig. 2a). However, when assessed by percent change from week 0, HFD-fed dogs showed a significant increase in body weight compared to ND-fed dogs starting at week 3 (HFD: 7.5 ± 9.3%, ND: − 0.5 ± 2.1%, p = 0.018), which continued for the duration of the study (Fig. 2c).

### Serum biochemistry after 12 weeks of ND or HFD

Chronic HFD feeding resulted in significantly higher cholesterol (235.7 ± 53.4 mg/dL, p = 0.019) and leptin levels (8.6 ± 7.6 ng/mL, p = 0.012) than ND feeding (187.9 ± 27.0 mg/dL, 1.6 ± 1.7 ng/mL, respectively, Table 1). Mean triglycerides appeared to be elevated in the HFD group (77.5 ± 71.5 mg/dL) compared to the ND group (54.1 ± 12.7 mg/dL), however this difference did not reach statistical significance (p = 0.310, Table 1). There was no effect of diet on ALP, ALT, AST, CK, adiponectin or NEFA levels (p > 0.05, Table 1).

**Table 1:** Serum biochemistry in dogs fed a ND (n = 10) or HFD (n = 11) for 12 weeks.

Serum biochemistry						
Parameter	Week 0			Week 12		
	ND	HFD	p-value	ND	HFD	p-value
ALP (IU/L)	58.9 ± 36.9	61.2 ± 23.2	0.869	63.6 ± 73.0	39.5 ± 16.4	0.333
ALT (IU/L)	53.2 ± 34.7	38.2 ± 11.5	0.219	50.7 ± 19.7	44.9 ± 14.9	0.461
AST (IU/L)	42.9 ± 12.6	39.5 ± 15.9	0.597	43.3 ± 8.9	41.0 ± 9.6	0.575
CK (IU/L)	258.5 ± 144.7	217.4 ± 120.3	0.490	213.1 ± 73.0	280.1 ± 168.1	0.249
Cholesterol (mg/dL)	214.8 ± 38.0	200.3 ± 28.8	0.341	187.9 ± 27.0	235.7 ± 53.4	0.019*
Triglycerides (mg/dL)	48.9 ± 14.7	49.9 ± 14.7	0.877	54.1 ± 12.7	77.5 ± 71.5	0.310
Leptin (ng/mL)	1.7 ± 1.0	5.1 ± 6.1	0.104	1.6 ± 1.7	8.6 ± 7.6	0.012*
Adiponectin (ug/mL)	8.7 ± 2.8	9.9 ± 3.7	0.429	11.3 ± 5.6	10.0 ± 4.8	0.570
NEFA (mmol/L)	0.7 ± 0.3	0.9 ± 0.5	0.378	0.9 ± 0.6	0.6 ± 0.2	0.156

All values shown are mean ± SD. *ALP* alkaline phosphatase, *ALT* alanine transaminase, *AST* aspartate aminotransferase, *CK* creatine kinase, *NEFA* non-esterified fatty acids. *p < 0.05; **p < 0.01; ***p < 0.001.

### Insulin sensitivity and beta cell compensation after 12 weeks of ND or HFD

**Figure 3:**
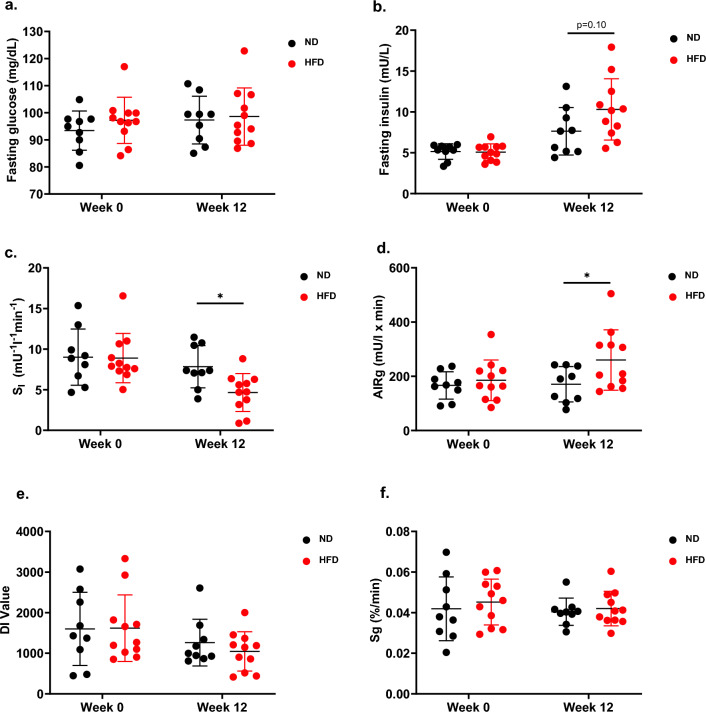
Chronic HFD feeding induces beta cell compensation and reduced insulin sensitivity. (**a**) Fasting glucose and (**b**) insulin concentrations prior to IVGTT in dogs fed a ND (n = 9) or HFD (n = 11) for 12 weeks. IVGTT measurements of (**c**) S_I,_, (**d**) AIR_G_, (**e**) DI and (**f**) S_G_ in dogs fed a ND (n = 9) or HFD (n = 11) for 12 weeks. All values shown are mean ± SD. *p < 0.05; **p < 0.01; ***p < 0.001.

After 12 weeks, fasting glucose remained stable in both the ND and HFD-fed groups. In contrast, fasting insulin was higher in HFD-fed dogs (10.3 ± 3.8 mIU/L) than ND-fed dogs (7.6 ± 2.9, p = 0.10, Fig. 3a,b), however this did not reach statistical significance.

At week 0, both ND and HFD groups had similar S_I_ values. However, by week 12, the HFD-fed group had significantly lower S_I_ values than dogs fed the ND (7.8 ± 2.6 [mU/l]^–1^ min^–1^ vs. 4.7 ± 2.3 [mU/l]^–1^ min^–1^, respectively, p = 0.012). The S_I_ values of the HFD-fed group decreased by 47% from week 0 levels (from 8.9 ± 3.0 [mU/l]^–1^ min^–1^ at week 0 to 4.7 ± 2.3 [mU/l]^–1^ min^–1^ at week 12), whereas it remained unchanged in the ND-fed group (Fig. 3c).

At week 0, both ND and HFD groups had similar AIR_G_ values. However, by week 12, AIR_G_ was significantly elevated in HFD-fed dogs relative to ND-fed dogs (260.2 ± 111.4 [mU/l]min vs. 170.7 ± 65.3 [mU/l]min, respectively; p = 0.039). The AIR_G_ of the HFD-fed group increased by 41% from week 0 levels (187.4 ± 78.7 [mU/l]min at week 0 vs. 260.2 ± 111.4 [mU/l]min at week 12) (Fig. 3d). DI and S_G_ were unchanged after 12 weeks of HFD feeding (Fig. 3e,f).

### Plasma lipid composition after 8 weeks of ND or HFD

Both dogs in the ND and HFD groups had similar plasma lipid composition profiles for all lipid species measured prior to study start at week 0 (p > 0.05, Table 2). Chronic HFD feeding resulted in elevations in plasma CE (p = 0.024), HCER (p < 0.001), LPC (p = 0.035), PC (p = 0.036), PE (p = 0.002) and SM (p = 0.001) compared to dogs in the ND group (Table 2).

**Table 2:** Plasma lipid species composition in dogs fed a ND (n = 10) or HFD (n = 11) for 8 weeks.

Lipid species (µM)	Week 0			Week 8		
	ND	HFD	p-value	ND	HFD	p-value
CER	4.3 ± 1.1	4.3 ± 0.6	0.961	4.6 ± 1.1	5.1 ± 0.9	0.214
CE	1545.9 ± 109.6	1545.3 ± 182.7	0.992	1486.8 ± 173.7	1771.2 ± 331.2	0.024*
DAG	7.9 ± 2.3	8.0 ± 2.9	0.935	7.3 ± 1.8	7.8 ± 2.7	0.585
DCER	1.7 ± 0.2	1.6 ± 0.2	0.528	1.6 ± 0.3	1.7 ± 0.2	0.430
HCER	1.2 ± 0.1	1.2 ± 0.2	0.682	1.1 ± 0.2	1.6 ± 0.3	< 0.001***
LCER	2.2 ± 0.5	2.1 ± 0.4	0.587	3.4 ± 0.7	3.9 ± 0.7	0.115
LPC	232.9 ± 16.4	228.8 ± 28.1	0.686	216.8 ± 22.9	245.2 ± 33.6	0.035*
LPE	7.1 ± 1.3	6.9 ± 1.1	0.699	6.6 ± 1.0	7.2 ± 1.1	0.205
MAG	2.4 ± 2.3	3.8 ± 2.9	0.225	5.4 ± 7.7	3.5 ± 3.1	0.488
PC	3650.3 ± 332.3	3742.3 ± 324.3	0.529	3513.0 ± 537.2	4141.2 ± 731.0	0.036*
PE	59.4 ± 8.1	61.9 ± 8.8	0.506	55.5 ± 6.3	70.0 ± 11.5	0.002**
PI	3.0 ± 1.2	2.6 ± 1.1	0.424	2.4 ± 0.7	3.1 ± 1.1	0.119
SM	325.1 ± 25.5	328.4 ± 32.7	0.799	313.6 ± 41.1	400.0 ± 62.3	0.001**
TAG	295.6 ± 85.4	292.5 ± 106.3	0.942	211.5 ± 39.8	252.1 ± 61.6	0.088

All values shown are mean ± SD.

*CER* Ceramides, *CE* cholesteryl esters, *DAG* diacylglycerol, *DCER* dihydroceramide, *HCER* hexosylceramide, *LCER* lactosylceramide, *LPC* lysophosphatidylcholine, *LPE* lysophosphatidylethanolamine, *MAG* monoacylglycerol, *PC* phosphatidylcholine, *PE* phosphatidylethanolamine, *PI* phosphatidylinositol, *SM* sphingomyelin, *TAG* triacylglycerol. *p < 0.05; **p < 0.01; ***p < 0.001.

### Fatty acid profiles after 12 weeks of ND or HFD

Both dogs in the ND and HFD groups had similar plasma fatty acid profiles in all fatty acid species measured prior to study start at week 0 (p > 0.05, Table 3). Chronic HFD feeding resulted in elevated total plasma fatty acids species 15:0, 16:0, 17:0, 18:0, 18:1, 18:2, 20:0, 20:1, 20:2, 20:3, 20:4, 22:0, 22:4 and aggregated FFA and SFA compared to dogs in the ND group (all p < 0.05, see Table 3 for p-values). Dogs in the HFD group also demonstrated significant reductions in plasma fatty acids 22:5 and 22:6 (all p < 0.05) and trending decrease in 20:5 compared to dogs in the ND group (p = 0.076, Table 3).

**Table 3:** Plasma fatty acid species composition in dogs fed a ND (n = 10) or HFD (n = 11) for 8 weeks.

Fatty acid quantification						
Fatty acid (µM)	Week 0			Week 8		
	ND	HFD	p-value	ND	HFD	p-value
Total [FA12:0]	0.8 ± 0.3	0.9 ± 0.5	0.669	1.6 ± 0.5	1.2 ± 0.5	0.092
Total [FA14:0]	24.6 ± 5.0	23.5 ± 6.4	0.661	30.1 ± 4.2	26.7 ± 6.0	0.140
Total [FA14:1]	2.3 ± 0.7	2.3 ± 1.2	0.959	2.2 ± 0.8	2.9 ± 1.8	0.254
Total [FA15:0]	9.1 ± 1.8	8.6 ± 3.0	0.655	9.5 ± 1.2	7.0 ± 1.2	< 0.001***
Total [FA16:0]	1742.1 ± 123.4	1760.3 ± 178.9	0.788	1643.8 ± 198.7	2211.1 ± 347.8	< 0.001***
Total [FA16:1]	139.9 ± 34.8	135.0 ± 25.8	0.716	134.3 ± 21.9	120.0 ± 22.9	0.160
Total [FA17:0]	36.2 ± 5.8	34.5 ± 3.3	0.412	41.4 ± 4.8	28.7 ± 4.2	< 0.001***
Total [FA18:0]	2438.3 ± 287.4	2525.2 ± 295.1	0.503	2325.1 ± 418.9	2796.7 ± 529.7	0.035*
Total [FA18:1]	1238.9 ± 121.2	1238.1 ± 125.5	0.989	1137.4 ± 106.7	1295.7 ± 181.9	0.025*
Total [FA18:2]	2135.1 ± 195.6	2173.7 ± 215.0	0.671	1867.1 ± 191.8	2268.6 ± 378.1	0.007**
Total [FA18:3]	66.5 ± 10.1	61.4 ± 16.6	0.401	51.1 ± 5.4	49.1 ± 10.9	0.600
Total [FA18:4]	0.4 ± 0.1	0.4 ± 0.1	0.149	1.8 ± 1.3	1.1 ± 0.7	0.157
Total [FA20:0]	28.3 ± 2.2	28.9 ± 1.6	0.554	26.1 ± 2.7	31.8 ± 5.0	0.004**
Total [FA20:1]	11.0 ± 1.1	11.3 ± 1.6	0.669	10.0 ± 1.3	12.0 ± 1.2	0.002**
Total [FA20:2]	23.7 ± 3.5	23.9 ± 2.4	0.867	20.0 ± 2.7	24.8 ± 3.4	0.002**
Total [FA20:3]	172.0 ± 43.3	169.5 ± 31.6	0.879	136.6 ± 37.3	181.9 ± 33.4	0.009**
Total [FA20:4]	1949.9 ± 258.9	1995.4 ± 264.3	0.695	1422.6 ± 293.5	1863.7 ± 412.5	0.011*
Total [FA20:5]	35.2 ± 6.3	30.7 ± 8.0	0.165	355.4 ± 199.8	213.8 ± 131.0	0.076
Total [FA22:0]	47.8 ± 5.1	49.2 ± 4.2	0.498	41.9 ± 6.6	54.3 ± 10.4	0.004**
Total [FA22:1]	12.6 ± 1.1	12.6 ± 1.0	0.965	11.0 ± 1.4	11.0 ± 1.9	0.970
Total [FA22:2]	0.8 ± 0.2	0.8 ± 0.2	0.747	0.7 ± 0.2	0.7 ± 0.1	0.939
Total [FA22:4]	77.4 ± 18.1	82.6 ± 22.1	0.561	25.9 ± 5.3	36.6 ± 10.6	0.010**
Total [FA22:5]	143.3 ± 21.5	144.3 ± 22.4	0.917	242.9 ± 47.1	170.4 ± 38.8	0.001**
Total [FA22:6]	51.5 ± 14.3	54.6 ± 12.8	0.615	231.2 ± 75.0	169.5 ± 54.6	0.048*
Total [FA24:0]	17.7 ± 1.8	17.3 ± 1.8	0.591	15.8 ± 2.0	15.6 ± 2.3	0.834
Total [FA24:1]	44.3 ± 4.0	43.8 ± 5.9	0.853	44.4 ± 6.5	43.6 ± 6.9	0.808
Total [FA26:0]	0.5 ± 0.0	0.5 ± 0.1	0.341	0.4 ± 0.1	0.5 ± 0.1	0.177
Total [FA26:1]	0.9 ± 0.1	0.9 ± 0.1	0.587	0.7 ± 0.1	0.8 ± 0.2	0.215
Σ SFA	4345.3 ± 396.4	4448.7 ± 401.4	0.560	4135.7 ± 583.0	5173.6 ± 884.1	0.005**
Σ MUFA	1449.9 ± 153.7	1444.0 ± 149.9	0.930	1340.0 ± 130.2	1486.1 ± 201.3	0.062
Σ PUFA	4655.9 ± 467.4	4737.2 ± 436.8	0.686	4355.2 ± 594.1	4980.3 ± 885.5	0.072
Σ FFA	7797.0 ± 696.1	7928.2 ± 689.6	0.670	7200.2 ± 836.5	8770.0 ± 1401.8	0.006**

All values shown are mean ± SD. *FFA* Free fatty acids, *SFA* saturated fatty acids, *MUFA* monounsaturated fatty acids, *PUFA* polyunsaturated fatty acids.

*p < 0.05; **p < 0.01; ***p < 0.001.

### Association between SFA levels, fasting insulin, insulin sensitivity and beta cell compensation

**Figure 4:**
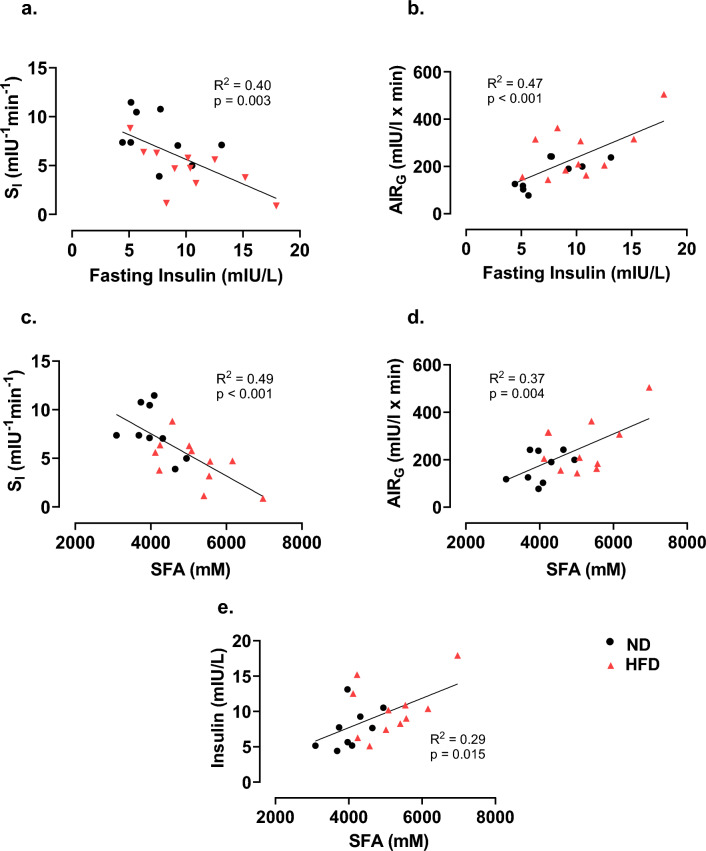
Fasting insulin and SFA are associated with insulin sensitivity. Correlations of (**a**) fasting insulin to insulin sensitivity S_I_, (**b**) fasting insulin to AIR_G_, (**c**) SFA to S_I_, (**d**) SFA to AIR_G_, and (**e**) SFA to fasting insulin in dogs after 12 weeks of HFD or ND feeding (n = 20).

Associations were assessed using the SFA values generated at week 8 and IVGTT parameters generated at week 12 in both ND and HFD dogs (n = 20). Pearson’s correlations demonstrated that fasting insulin is negatively associated with S_I_ (R^2^ = 0.40) and positively associated with AIR_G_ (R^2^ = 0.47) and aggregated SFA concentrations (R^2^ = 0.29) (all p ≤ 0.015, Fig. 4a,b,e). Aggregated SFA concentrations were also negatively associated with S_I_ (R^2^ = 0.49) and positively associated with AIR_G_ (R^2^ = 0.37) in all dogs (all p < 0.01, Fig. 4c,d).

## Discussion

In this investigation we aimed to characterize the changes in the plasma lipidome resulting from chronic HFD feeding and determine if these changes were associated with the deterioration of insulin sensitivity and beta cell compensation. Increased concentrations of deleterious lipid species and fatty acids, such as sphingolipids and long chain saturated fatty acids, have been associated with visceral adipose mass and the development of metabolic disease in humans^22,23^, while the accumulation of certain PUFA species has been shown to be metabolically beneficial^24^. The impact of these lipid species are well described in metabolically stressed rodents and humans, however little is known about the plasma lipidome in dogs presented with a metabolic stressor such as HFD.

Recapitulating previous investigations, chronic HFD feeding in dogs led to hyperphagia and increased body weight, likely due to an increase in total adiposity. This apparent adipose expansion is likely due to the increased levels of leptin, an adipokine which has a known positive relationship with increased abdominal adipose tissue mass and a hallmark of metabolic disease^25^. Additionally, this study demonstrated HFD feeding dogs induces elevations in clinically measured total triglycerides and cholesterol, suggesting the presence of dyslipidemia, which has been previously reported^16^. The serum biochemistry of dogs fed a HFD in this study is similar to previous investigations in rodents, while mirroring aspects of metabolic disease in human populations^26–29^.

Our study is also consistent with previous investigations describing the effects of HFD feeding on insulin sensitivity and beta cell compensation. Here we show a similar phenotype, albeit using a different macronutrient composition HFD, whereby insulin sensitivity is compromised and hyperinsulinemic compensation from the beta cells is required to maintain glycemic control^12,14,30^. This phenotype is analogous to HFD models in rodents and characteristic of metabolic disease and insulin resistance in humans^31,32^.

However, unlike rodents and humans, the hyperinsulinemic compensation and reduced insulin sensitivity in the HFD-fed group occurs without impacting glycemia. This innate ability for dogs to compensate for blunted peripheral insulin sensitivity by continually producing additional insulin in order to maintain euglycemia, even in the presence of metabolic stressors, has previously been reported in several models of metabolic dysfunction in dogs^33^. Although glycemia in dogs is highly regulated, the presence of continual hyperinsulinemic compensation and peripheral insulin resistant phenotypes may have deleterious effects as seen in other species^34,35^. Most notably, metabolically stressed obese dogs have reduced lifespans, a phenotype also present in human populations^36,37^. Additional studies are needed to further define the clinical impact of hyperinsulinemia and impaired insulin sensitivity in dogs.

In healthy dogs, fasting hyperinsulinemia has been associated with impaired insulin sensitivity when measured by hyperinsulinemic euglycemic clamp^38^. Our data recapitulate the association between fasting insulin and insulin sensitivity in all dogs assessed, regardless of diet. Interestingly, we further identify that fasting hyperinsulinemia is associated with commensurate beta cell compensation, augmenting the previous findings that fasting insulin, not fasting glycemia, is indicative of the insulin sensitivity state in dogs^33^.

While the effects of HFD on body weight, fasting biochemistry and insulin sensitivity have been previously described in dogs^13^, a considerable knowledge gap exists regarding the composition of circulating lipid species and how they impact metabolic function. Our data reveal that chronic consumption of HFD leads to significant elevations in the plasma lipid species CE, HCER, LPC, PE, PC and SM. The elevation of many of these lipids are notable, namely HCER, PC and SM, due to their roles as biological intermediates in the formation of ceramides and other sphingolipids. The increased abundance of these lipid species has been previously implicated as deleterious actors in the development of tissue lipotoxicity and have been associated with impaired insulin action and sensitivity^39^. Bidirectionality has also been established by *Warshauser *et al. wherein reducing levels of HCER and other ceramides pharmacologically improved HbA1C, insulin sensitivity and beta cell function in patients with existing MetS^40^. More studies are needed to determine if a simple correlative or causal relationship exists between these lipid species and impaired insulin sensitivity in both dogs and humans.

Plasma fatty acid concentrations and their physiological role in the development of insulin resistance, MetS and T2D have been investigated for decades in humans and rodents^17,41^. Of note, *Kang *et al. demonstrate that increases in visceral adipose mass, not subcutaneous adipose mass, are associated with elevated fatty acid concentrations in humans^42^. Our analysis of fatty acid profiles in dogs revealed a significant elevation in fatty acid species that have been directly implicated in the deterioration of insulin sensitivity, such as palmitic and stearic acid^43,44^. Furthermore, HFD feeding elevated nearly all SFA species and associated 18:1 (oleic acid) and 18:2 (linoleic acid), while significantly reducing the abundance of DPA and DHA—2 fatty acids associated with improved insulin sensitivity^45^.

Two groups of aggregated fatty acids, FFA and SFA, were also elevated with HFD feeding while no change was observed to aggregated MUFA and PUFA pools. The presence of persistently elevated FFA and SFA concentrations has been linked to the development of ectopic lipid deposits, inflammation, lipotoxicity and impaired insulin signaling in other species^17^. These changes in fatty acids, along with the previously described changes in plasma lipid species composition, suggests that HFD induces deleterious changes in the lipidome which may elicit metabolic consequences on peripheral tissues, such as the skeletal muscle and liver^46,47^.

As previously mentioned, nearly every species of SFA was significantly elevated as a result of the HFD feeding and has been associated with obesity and T2D in humans^17,19^. Therefore, we examined the relationship between SFA concentrations, insulin and insulin sensitivity metrics to determine if an association could be established. We show that as SFA concentrations increase, insulin sensitivity is deteriorated, while AIR_G_ is increased to compensate. This finding is supported by previous studies which infused a fatty acid cocktail consisting of the most abundant fatty acids present in our HFD model and caused impairments in the whole body and peripheral insulin sensitivity of dogs^19^.

Interestingly, the association between SFA concentrations and insulin sensitivity was stronger than its association with fasting insulin (SFA R^2^: 0.49; fasting insulin R^2^: 0.40). Conversely, beta cell compensation is associated more strongly with fasting insulin than SFA concentrations. These data suggest that SFA concentrations may be a useful predictor of insulin sensitivity, regardless of dietary status in dogs. Further investigations are needed to determine if SFA concentrations are predictive of metabolic health, specifically insulin sensitivity, in real world companion dog populations.

The present study had several limitations. Dogs in the HFD group were presented with a double daily ration of food, meanwhile dogs in the ND group were presented with their normal single ration. Due the ND and HFD groups seeing different total quantities of food, it is difficult to decouple the metabolic effects of elevated fat content in the HFD from simple hyperphagic overnutrition. Additionally, tissue specific gene expression and protein changes, which greatly impact substrate utilization, were not investigated and therefore we are unable to make conclusions about the molecular impact to peripheral tissue.

## Conclusion

These results demonstrate that chronic HFD feeding leads to deleterious changes in the plasma lipidome, fatty acid species composition, hyperinsulinemic compensation and impaired insulin sensitivity in dogs. Furthermore, we demonstrated that HFD feeding elevated nearly every SFA species while reducing the abundance of metabolically beneficial species DPA and DHA. Elevations in SFA concentrations were associated with deteriorated insulin sensitivity, which identifies, for the first time, the detrimental changes in plasma fatty acid species and the development of metabolic dysfunction in dogs.

### Supplementary Information

Supplementary Tables.

## Data Availability

All data available by request from authors.
