# Supplementary material for: Saturated fatty acid concentrations are predictive of insulin sensitivity and beta cell compensation in dogs
Source: Sci Rep. 2024 Jun 2;14:12639. doi: 10.1038/s41598-024-63373-5 (PMC11144705; doi:10.1038/s41598-024-63373-5)
Supplement: Supplementary file 1 — Supplementary Tables. [file 41598_2024_63373_MOESM1_ESM.docx]

1. **VetBrands Premium (dry)**

| **Nutrient/ingredient** | **Amount** | **Unit** | **Kcal/kg** | **Percent of kcal** |
| --- | --- | --- | --- | --- |
| Crude protein | 220 | g/kg (min) | 880 | 39% |
| Moisture | 100 | g/kg (max) | 0 | 0% |
| Crude fat | 110 | g/kg (min) | 990 | 43% |
| Crude fibre | 35 | g/kg (max) | 140 | 6% |
| Crude ash | 80 | g/kg (max) | 0 | 0% |
| Phosphorus | 12 | g/kg (min) | 0 | 0% |
| Calcium | 16 | g/kg (max) | 0 | 0% |
| Omega 6: Omega 3 | 7-10 : 1 |  |  |  |
| Linoleic Acid | 30 | g/kg (min) | 270 | 12% |
| Glucosamine | 250 | mg/kg (min) | 0 | 0% |
| Glucans | 135 | mg/kg (min) | 0 | 0% |

1. **Purina Husky Adult (wet)**

| **Nutrient/ingredient** | **Amount** | **Unit** | **Kcal/kg** | **Percent of kcal** |
| --- | --- | --- | --- | --- |
| Crude protein | 50 | g/kg (min) | 200 | 48% |
| Moisture | 820 | g/kg (max) | 0 | 0% |
| Crude fat | 20 | g/kg (min) | 180 | 43% |
| Crude fibre | 9 | g/kg (max) | 36 | 9% |
| Crude ash | 25 | g/kg (max) | 0 | 0% |

1. **Pork Lard (wet)**

| **Nutrient/ingredient** | **Amount** | **Unit** | **Kcal/kg** | **Percent of kcal** |
| --- | --- | --- | --- | --- |
| Crude fat | -- | g/kg (min) | 9000 | 100% |

Supplement Table 1: A) VetBrands Premium Dog Food nutrient composition used as dry normal diet. B) Purina Husky Adult nutrient composition used in the 66% and 74% fat diets C) Pork lard nutrient composition used in the 66% and 74% fat diets.

| 1. **Diet Macronutrient Composition** | | | |
| --- | --- | --- | --- |
| Diet | Protein (%) | Carbohydrate (%) | Fat (%) |
| ND (55%) | 39 | 6 | 55 |
| HFD (66%) | 29 | 5 | 66 |
| HFD (74%) | 22 | 4 | 74 |

| 1. **Diet Mixtures** | | | |
| --- | --- | --- | --- |
| Diet | VetBrands Premium (%) | Purina Husky (%) | Pork Lard (%) |
| ND (55%) | 100.0 | 0.0 | 0 |
| HFD (66%) | 67.1 | 26.8 | 6.0 |
| HFD (74%) | 62.5 | 25.0 | 12.5 |

Supplement Table 2: A) Macronutrient composition of all diets B) Percentages of each diet used to achieve macronutrient composition.

| **Parameter** | **HFD** (n=11) | **ND** (n=10) | **p-value** |
| --- | --- | --- | --- |
| **Body Weight** |  |  | 0.933 |
| Mean (SD) | 17.38 (4.49) | 17.52 (2.77) |  |
| Median (IQR) | 15.60 (14.10, 20.05) | 17.05 (15.30, 19.30) |  |
| Range | 13.40, 25.00 | 14.70, 23.10 |  |
| **BCS** |  |  | 0.509^1^ |
| Mean (SD) | 5.09 (0.30) | 5.20 (0.42) |  |
| Median (IQR) | 5.00 (5.00, 5.00) | 5.00 (5.00, 5.00) |  |
| Range | 5.00, 6.00 | 5.00, 6.00 |  |
| **Castrated** | 4 (36%) | 5 (50%) | 0.670^2^ |
| **Mix or Beagle** |  |  | 1.000^2^ |
| Beagle | 3 (27%) | 3 (30%) |  |
| Mixed | 8 (73%) | 7 (70%) |  |
| ^1^ Welch Two Sample t-test; ^2^ Fisher’s exact test SD = Standard Deviation; IQR = Interquartile range (Q1, Q3) | | | |

Supplement Table 3: Pre-study enrollment descriptive statistics.

| **Class** | **Number of Species** |
| --- | --- |
| Ceramide (CER) | 12 |
| Cholesteryl Esters (CE) | 26 |
| Diacylglycerols (DAG) | 58 |
| Dihydroceramide (DCER) | 12 |
| Hexosylceramide (HCER) | 12 |
| Lactosylceramide (LCER) | 12 |
| Lysophosphatidylcholine (LPC) | 26 |
| Lysophosphatidylethanolamine (LPE) | 26 |
| Monoacylglycerol (MAG) | 26 |
| Phosphatidylcholine (PC) | 140 |
| Phosphatidylethanolamine (PE) | 216 |
| Phosphatidylinositol (PI) | 28 |
| Sphingomyelin (SM) | 12 |
| Triacylglycerols (TAG) | 519 |

**Supplement Table 4:** Lipid species quantified in the Complex Lipid Targeted Panel from Metabolon Inc.
